# Immune Thrombocytopenic Purpura Secondary to Cytomegalovirus (CMV) Infection: A Clinical Case

**DOI:** 10.7759/cureus.75334

**Published:** 2024-12-08

**Authors:** Francisca Nunes Silva, Cláudia Batista Rosa, Bárbara Vasconcelos, Catarina Teixeira de Jesus, Cátia Gonçalves, Carla Gonçalves Rocha, Helena Fragoeiro, João Catanho, Roberta Valladares, Sérgio Freitas

**Affiliations:** 1 General and Family Medicine, Câmara de Lobos Health Center, Serviço de Saúde da Região Autónoma da Madeira, Entidade Pública Empresarial da Região Autónoma da Madeira (SESARAM, EPERAM), Câmara de Lobos, PRT; 2 General and Family Medicine, Caniço Health Center, Serviço de Saúde da Região Autónoma da Madeira, Entidade Pública Empresarial da Região Autónoma da Madeira (SESARAM, EPERAM), Santa Cruz, PRT

**Keywords:** cytomegalovirus, epistaxis, immune thrombocytopenic purpura, petechiae, thrombocytopenia

## Abstract

Immune thrombocytopenic purpura (ITP) is an autoimmune condition characterized by a reduced platelet count due to enhanced peripheral destruction and impaired platelet production. While thrombocytopenia is a well-documented complication of various viral infections, cytomegalovirus (CMV), a member of the Herpesviridae family, is primarily associated with infections in immunocompromised patients and is rarely implicated in causing severe thrombocytopenia in immunocompetent patients. This article aims to highlight the importance of considering CMV as a significant etiological factor in ITP, particularly in cases of asymptomatic thrombocytopenia.

A 34-year-old male presented to the emergency department with a one-week history of progressively worsening erythematous macules on the lower extremities, extending proximally, accompanied by spontaneous ecchymoses, ulcerative and hemorrhagic aphthous lesions in the oral cavity and recurrent episodes of epistaxis. Physical examination revealed petechiae on the ear pavilions, face, trunk, back, upper limbs, abdomen and lower limbs. Intraorally, multiple ulcers and violaceous lesions were observed on the buccal mucosa and tonsillar regions. Additionally, the patient exhibited a localized area of ecchymosis with a central crusted lesion on the right lower leg. Serologic testing for CMV was positive and targeted antiviral therapy was initiated.

CMV infection can lead to severe thrombocytopenia in otherwise healthy adults, despite diagnostic challenges. These challenges arise due to the low incidence of severe CMV disease, its diverse clinical manifestations, and its ability to mimic symptoms of other common illnesses. In cases of ITP that resist standard treatments, antiviral therapy may be necessary and should be promptly initiated if CMV infection is confirmed.

CMV infection should be included in the differential diagnosis for severe thrombocytopenia in healthy adults, especially following a recent viral infection.

## Introduction

Immune thrombocytopenic purpura (ITP) is a disorder of autoimmune nature marked by the destruction of peripheral blood platelets or the suppression of megakaryopoiesis in the bone marrow. This occurs due to autoantibodies targeting surface antigens on platelets and megakaryocytes. Consequently, the platelet count drops below 100 × 10^9/L, often accompanied by systemic complications such as gastrointestinal bleeding, petechiae, and purpura [1].

ITP is the most common presentation of thrombocytopenia affecting otherwise asymptomatic adults. Secondary ITP, arising from an underlying condition, is a diagnosis of exclusion, which is crucial for determining effective treatment strategies [2]. It may be caused by drugs, immunodeficiency states, antiphospholipid syndrome, lymphoproliferative disorders, or infectious diseases such as those caused by cytomegalovirus (CMV), Helicobacter pylori, human immunodeficiency virus (HIV), and hepatitis C virus [3]. Secondary thrombocytopenia due to CMV infection is relatively common in immunocompromised, but documented cases in immunocompetent adults remain rare [4].

Cytomegalovirus is a virus of the herpesvirus family, and its immunoglobulin G (IgG) antibodies were found in around 60% of the adult population in developed countries [5]. While thrombocytopenia is a recognized complication of various viral infections, such as those caused by rubella, Epstein-Barr virus, mumps, varicella, HIV, and hepatitis, severe thrombocytopenia due to CMV is rare in immunocompetent patients [6].

Efficient CMV dissemination and infection of different cell types are facilitated by broad cellular tropism, by interactions between viral gH/gL glycoprotein complexes and surface receptors on host cells. Recently, the combined knowledge of immunology and molecular biology has suggested that CMV infection equally triggers and perpetuates ITP in both immunocompetent and immunosuppressed patients [7].

The pathophysiology of thrombocytopenia in conjunction with CMV has not been completely explained but there are several theoretical reasons: direct cytotoxicity of CMV to hematopoietic cells/impairment of bone marrow stromal function; immune-mediated destruction of infected cells; and antibody-mediated (non-specific autoantibodies) destruction of platelets induced by CMV infection [8,9].

Previous reports have demonstrated that immature hematopoietic cells can be infected with CMV. They have shown that the completion of the viral life cycle is dependent on myeloid differentiation and that only mature myeloid and monocytic cells can support CMV infection. Additionally, it has been speculated that CMV can also infect and endure in mature megakaryocytic (MK) cells [8,9].

Adam S Levy et al. have found that CMV-infected CD34 progenitors and stem cells can survive the initial infection and differentiate into mature MK cells. On the differentiation of megakaryocytic precursors, CMV can complete its life cycle and promote the release of more viruses [10].

Including CMV infection in the differential diagnosis for thrombocytopenia can support timely diagnosis and appropriate treatment, thereby helping prevent complications, reducing the need for invasive tests, and avoiding unnecessary healthcare costs associated with bleeding and other procedural complications [6].

Steroids and intravenous immunoglobulin (IVIG) are the treatment of choice for ITP. CMV-thrombocytopenia, however, is reported to be less responsive to standard therapies and may require antiviral treatment, highlighting the importance of primary infection treatment before generically treating ITP [4]. This is aligned with the case reported by Bessy S. Flores-Chang et al. in which the thrombocytopenia and patient’s symptoms were not significantly improved with methylprednisolone and IVIG, but only upon discovery of underlying CMG infection and valganciclovir started on regiment of 900 mg PO BID, did the patient improve clinically and analytically [2]. Furthermore, the case series and literature review of Tamir Shragai et al., which included 23 articles reported that in CMV-associated ITP, the response efficacy increased by almost 50% when treating CMV-ITP with antivirals anti-CMV opposed to first-line agents such as steroids [3]. Another case published this year by Emma Roberts et al. also highlights that in immunocompetent patients presenting with ITP and CMV infection, valganciclovir leads to complete recovery in two weeks' time [11]. Other 2024 reviews underlined cases where oral valganciclovir led to complete recovery in immunocompetent patients with primary CMV infection complicated by ITP [12].

This clinical case delves into the presentation, diagnosis, and management of post-viral thrombocytopenia secondary to CMV infection, pointing out the importance of considering CMV as a possible cause of thrombocytopenia, especially in patients with compatible symptoms and risk factors [2].

This article aims to highlight the need to recognize CMV as an important etiology in cases of otherwise asymptomatic thrombocytopenia and emphasize the need for heightened clinical awareness as well as a multidisciplinary approach for diagnosing and treating post-viral thrombocytopenia and ensuring optimal patient outcomes in the context of CMV infection [2].

## Case presentation

A 34-year-old male with a medical history of dyslipidemia and obesity, not on regular medication and with up-to-date vaccination, without smoking habits, drug use, or known drug allergies, presented to the Emergency Department with a one-week history of progressive erythematous macules on the lower extremities, associated with spontaneous ecchymoses and one-day evolution of ulcerated, intermittently bleeding aphthous lesions in the oral cavity, along with multiple episodes of spontaneous epistaxis. The patient reported having recently experienced symptoms of a "cold", and frequent mosquito bites prior to the onset of his current condition.

He denied pain pruritus, fever, night sweats, weight loss, cough, vomiting, chest pain, dyspnea or penile discharge, recent travel, or contact with rats or ticks. On physical examination, the patient was eupneic at rest with an oxygen saturation of 97% on room air, blood pressure of 145/82 mmHg, heart rate of 97 bpm, and a body temperature of 36.9 ºC. He was acyanotic, with hydrated mucous membranes. Skin examination revealed petechiae on the face, trunk, back, upper limbs, abdomen, and lower limbs. Intraoral examination showed: ulcers and violaceous lesions in the buccal mucosa and tonsils. Cardiac and pulmonary auscultation were normal, and the abdomen was soft with normal bowel sounds, no palpable masses or organomegaly, and no tenderness on palpation.

The lower extremities showed no edema or signs of deep vein thrombosis but several confluent petechial lesions were noted. A rounded ecchymotic area with a central crusted lesion was observed on the inner right leg. Laboratory studies were requested, including a peripheral blood smear (PBS), serologies for Epstein-Barr virus, CMV, Parvovirus B19, dengue, and Rickettsia spp, as well as rapid antigen test (RAT) for influenza A, influenza B, and COVID-19.

Hematological analysis revealed severe thrombocytopenia <3,000 platelets/μL and the presence of schizocytes on PBS. RATs for influenza A, B, and COVID-19 were negative. Serologies were negative for all tested pathogens except for CMV, which showed positive IgG, positive IgM, and a CMV-DNA level of 490 copies/mL.

A diagnosis of post-viral thrombocytopenia secondary to symptomatic CMV infection was made. The patient was admitted to the observation unit and received 1000 mg/day of intravenous methylprednisolone and 30 mg of intravenous immunoglobulin (IVIG). After two days, he was transferred to the hematology unit, with a rising platelet count of 5,000/μL. He was started on prednisolone 40 mg twice daily and valganciclovir 900 mg every 12 h, in addition to completing a five-day course of IVIG.

The patient was discharged three days after admission to the hematology ward, clinically stable, on a regimen of prednisolone 80 mg/day, pantoprazole 20 mg/day, and valganciclovir 900 mg every 12 h. During follow-up at the outpatient clinic 10 days post-discharge, the patient's CMV-DNA remained detectable but had decreased to <250 copies/mL, and his platelet count had improved to 150,000/μL. A gradual weaning of prednisolone was initiated, starting at 60 mg/day. One month after discharge CMV-DNA was undetectable, and the platelet count had normalized to 242,000/μL. Valganciclovir was then reduced to prophylactic doses (900 mg once daily) for another two months. The patient continued a slow taper of prednisolone for one more month, achieving complete clinical and laboratorial resolution. No further symptoms occurred and a one-year follow-up was scheduled at the hematology outpatient clinic.

**Table 1:** Laboratory test results for viral and platelet assessment DNA - deoxyribonucleic Acid; EBV - Epstein-Barr vírus; ELISA - enzyme-linked immunosorbent assay; IgG - immunoglobulin G; IgM - immunoglobulin M; HIV - human immunodeficiency virus; PCR - polymerase chain reaction; RAT - rapid antigen test; RNA - ribonucleic acid; ^1^Laboratory cut-offs not provided; ^a^Combined test for influenza A, influenza B, and COVID-19.

Laboratory Tests	Result	Unit	Reference values
Platelet count	<3.0	1000/µL	144-440
EBV-EBNA IgG antibody	9.3	S/CO	Negative <0.50
			Inconclusive 0.50 - 1.00
			Positive ≥1.00
EBV-VCA IgG antibody	35.5	S/CO	Negative <0.75
			Inconclusive 0.75 - 1.00
			Positive ≥1.00
EBV-VCA IgM antibody	0.6	S/CO	Negative <0.50
			Inconclusive 0.50 - 1.00
			Positive ≥1.00
Parvovirus B19 IgG antibody (ELISA)	Positive	---	Negative^1^
			Inconclusive^1^
			Positive^1^
Parvovirus B19 IgM antibody (ELISA)	Negative	---	Negative^1^
			Inconclusive^1^
			Positive^1^
Cytomegalovirus IgG antibody	15.3	UA/mL	Negative <6.0
			Inconclusive 6.0 - 15.0
			Positive ≥15.0
Cytomegalovirus IgG antibody avidity	18.0%	%	Low avidity <50
			Indeterminate avidity 50 - 59,9
			High avidity ≥ 60
Cytomegalovirus IgM antibody	6.13	S/CO	Negative < 0.85
			Inconclusive 0.85 – 1.00
			Positive ≥1.00
Dengue IgG antibody	Negative	---	Negative^1^
			Inconclusive^1^
			Positive^1^
Dengue IgM antibody	Inconclusive	---	Negative^1^
			Inconclusive^1^
			Positive^1^
Rickettsia spp IgG antibody	Negative	---	Negative^1^
			Inconclusive^1^
			Positive^1^
Rickettsia spp IgM antibody	Inconclusive	---	Negative^1^
			Inconclusive^1^
			Positive^1^
DNA cytomegalovirus (PCR)	Positive: 490 copies/mL		Negative when no copies are found
			Positive when DNA copies are found, although it might not be measurable <250 copies
RNA Dengue virus (PCR)	Negative		Negative^1^
			Positive^1^
RAT - Respiratory viral panel^a^	Negative: pink-to-purple C line	---	Negative - pink-to-purple C line
			Positive - pink-to-purple C line + pink-to-purple T line

## Discussion

This case report underscores the importance of considering CMV infection as a cause of severe thrombocytopenia in otherwise healthy adults. The underlying mechanisms may involve direct cytotoxic effects of CMV on hematopoietic cells and immune-mediated destruction of infected cells by means of autoantibodies, ultimately leading to antibody-mediated platelet destruction. Diagnosing CMV infection in immunocompetent individuals poses a challenge due to factors such as the low incidence of severe CMV disease, its broad tissue tropism, and the wide range of clinical manifestations that may mimic those of other diseases contributing to diagnostic confusion and delays. Recognizing CMV as a differential diagnosis in otherwise healthy patients with thrombocytopenia can help prevent unnecessary invasive procedures such as bone marrow examinations and lymph node biopsies. Noninvasive diagnostic tools, including CMV-PCR, anti-CMV IgG avidity testing, urinary antigen detection, blood smear morphology, and viral culture, are often sufficient for diagnosing CMV infection and can also be valuable in monitoring treatment effectiveness.

Prompt treatment of CMV-induced thrombocytopenia is essential, and therapeutic options include corticosteroids, intravenous immunoglobulins, vincristine, splenectomy, and antiviral agents such as ganciclovir or valganciclovir. While antiviral therapy is first-line for severe CMV infection in immunodeficient adults, its benefit in immunocompetent patients remains unclear. Differentiating between CMV-induced thrombocytopenia and CMV-associated secondary ITP can be challenging, but antiviral therapy may be necessary in cases of ITP unresponsive to standard treatments when CMV infection is present.

Careful consideration of patient-specific factors and disease severity is crucial to thoughtfully assess the choice of suitable drugs and the optimal timing to start the treatment.

This case report highlights the importance of considering CMV infection as a potential cause of severe thrombocytopenia in otherwise healthy adults.

## Conclusions

In cases of severe thrombocytopenia in otherwise healthy adults, particularly following a recent viral infection, CMV infection should be investigated. Although spontaneous resolution is possible, ensuring timely and appropriate treatment remains crucial to improve the outcome and decrease the disease burden.
